# Style Transfer of Chinese Wuhu Iron Paintings Using Hierarchical Visual Transformer

**DOI:** 10.3390/s24248103

**Published:** 2024-12-19

**Authors:** Yuying Zhou, Yao Ren, Chao Wu, Minglong Xue

**Affiliations:** 1 Academy of Art and Design, Anhui University of Technology, Ma’anshan 243002, China; wuqishang008@outlook.com; 2 Engineeing Training Center, Nanjing Vocational University of Industry Technology, Nanjing 210023, China; 2021101304@niit.edu.cn; 3 College of Computer Science and Engineering, Chongqing University of Technology, Chongqing 400054, China; xueml@cqut.edu.cn

**Keywords:** Chinese Wuhu iron paintings, style transfer, feature symmetry, content correction module

## Abstract

Within the domain of traditional art, Chinese Wuhu Iron Painting distinguishes itself through its distinctive craftsmanship, aesthetic expressiveness, and choice of materials, presenting a formidable challenge in the arena of stylistic transformation. This paper introduces an innovative Hierarchical Visual Transformer (HVT) framework aimed at achieving effectiveness and precision in the style transfer of Wuhu Iron Paintings. The study begins with an in-depth analysis of the artistic style of Wuhu Iron Paintings, extracting key stylistic elements that meet technical requirements for style conversion. Furthermore, in response to the unique artistic characteristics of Wuhu Iron Paintings, this research constructs a multi-layered network structure capable of effectively capturing and parsing style and content features. Building on this, we have designed an Efficient Local Attention Decoder (ELA-Decoder) that adaptively decodes the style and content features through correlation, significantly enhancing the length dependency of local and global information. Additionally, this paper proposes a Content Correction Module (CCM) to eliminate redundant features generated during the style transfer process, further optimizing the migration results. In light of the scarcity of existing datasets for Wuhu Iron Paintings, this study also collects and constructs a dedicated dataset for the style transfer of Wuhu Iron Paintings. Our method achieves optimal performance in terms of loss metrics, with a reduction of at least 4% in style loss and 5% in content loss compared to other advanced methods. Moreover, expert evaluations were conducted to validate the effectiveness of our approach, and the results show that our method received the highest number of votes, further demonstrating its superiority.

## 1. Introduction

Deep learning is widely used in computer vision [1,2,3,4], computer graphics [5,6,7,8], and other fields. When the field of image generation is developing at a high speed, Image Style Transfer is also widely noticed by researchers. Image Style Transfer aims to transfer artistic features from style images to content images, thus bringing interesting visual perception effects for the delivery of artworks. Also, Gatys et al. [9] first proposed that correlation between features can be used as a basis for accurate style transfer and texture synthesis, thus developing convolutional neural networks (CNNs) for style transfers.

Along with the research, Image Style Transfer methods have evolved from the traditional [10] to the iterative paradigm [11] and feed-forward methods [12,13]. In addition, to address the problem of style bias in most images, the Universal Image Style Transfer (UST) has also gained significant development [14,15,16]. Based on this, innovations in various style Transfer methods have also been stimulated, such as the emergence of innovative approaches such as the Flow-based ArtFlow [17] and comparative learning-based approaches [18,19].

**Figure 1:**
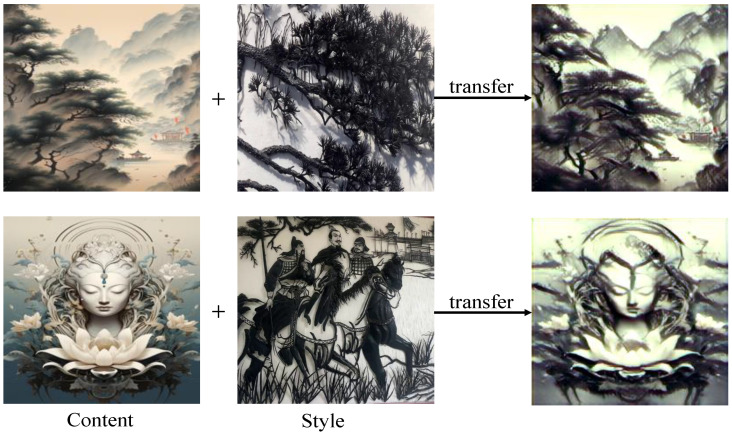
This figure shows the results of our method to convert the content image to the Wuhu Iron Paintings style. Due to the rich lines, layers, and unique texture of Wuhu Iron Paintings, the current technical solutions have not achieved the desired effect.

Despite the great progress in related research, most of the current approaches focus on the balanced conversion of multiple styles, or are specific to Western paintings, as all abstract paintings tend to be similar in style. However, unlike the scientific perspective in the West, Chinese Wuhu Iron Painting, as one of the traditional Chinese arts, has a certain concept of pictorial perspective, which is an idealistic feeling that permits one to depict what is beyond what is visible to the naked eye. In particular, the artistic three-dimensionality, multi-layered interspersion, texture expression, and presentation techniques of Chinese Wuhu Iron Paintings (as shown in Figure 1) further increase the difficulty of symmetry of features in style transfer, and the current state-of-the-art methods do not lead to satisfactory visual effects. Specifically, current methods do not pay good attention to global and local relationships, resulting in redundant information that damages the view when decoding features. Therefore, due to the consideration of computational resources and the current excellent performance of Hierarchical Vision Transformer in the field of image style migration, we further explored the two perspectives of decoder and removing redundant features.

In this paper, based on the above challenges, we propose a Hierarchical Visual Transformer based on the Wuhu Iron Painting style transfer network, which facilitates features to be symmetrized during style transfer, referred to in [16]. Specifically, inspired by [16], we rely on the hierarchical visual transformer’s multi-level windowed attention to efficiently capture content and style images’ local and global features. Subsequently, we designed the Efficient Local Attention Decoder (ELA-Decoder) to correlatively and adaptively decode stylistic features and content features through effective local attention to enhance the long and short-term dependencies between local and global information. Meanwhile, to further ensure the fidelity and friendliness of the visual perception of the transfer results, we design a content correction module (CCM) using a residual dense architecture to achieve the elimination of redundant features from the transfer results to obtain visually oriented transformation effects. In addition, due to the lack of an existing benchmark dataset of Chinese Wuhu Iron Paintings and also to further demonstrate the validity of the designed network, we first adopted the field survey method to collect a large number of original pictures of Wuhu Iron Paintings, and constructed the dataset of Wuhu Iron Paintings by classifying and processing the pictures; moreover, we combined the stylistic conversion practice of many types of pictures and the expert scoring method to evaluate the results qualitatively and quantitatively, to test and validate the effectiveness of the proposed method.

Our main contributions are summarized below:

- We propose a new network that achieves reliable feature encoding by relying on short and long-term modeling of content features and stylistic features with a hierarchical visual transformer and effective style transfer of Chinese Wuhu Iron Paintings with a designed attentional decoder.
- We further designed a content correction module that effectively captures redundant features and noise for rejection using a residual dense architecture to ensure the visual fidelity and friendliness of the migrated images.
- We collected a dataset of Iron Paintings from Wuhu, China, and evaluated it qualitatively and quantitatively to verify the validity of our method.

## 2. Related Work

### 2.1. Chinese Wuhu Iron Paintings

#### 2.1.1. Analysis of the Artistic Characteristics of Chinese Wuhu Iron Paintings

As a treasure of traditional Chinese arts and crafts, Wuhu Iron Paintings show their unique charm [20,21,22,23] in many dimensions such as modeling, theme, color, craftsmanship, and culture. In terms of modeling, Wuhu Iron Paintings are influenced by the characteristics of materials [24], taking iron as raw material and shaping iron sheets and wires through forging and other processes to create diversified shapes, which are characterized by smooth lines, strong three-dimensionality, and clear hierarchy. In the choice of themes, Wuhu Iron Paintings are often created with natural scenery, stories of people, and scenes of life, especially landscapes, plums, orchids, bamboo, chrysanthemums, etc. These themes also reflect the love of local people for nature. In terms of color, the traditional Wuhu Iron Paintings are black and white. With the development of the times, the current ones also include gold, green, and other colors, but most of the Iron Paintings, in terms of the choice of color, are still mainly black and white. In terms of technology, Wuhu Iron Paintings take cooked iron as raw material, combine forging as the main production technology, and integrate drilling, lifting, pressing, welding, filing, chiseling, and other technologies, which makes Wuhu Iron Paintings more accurate and delicate in the shaping of things. In terms of cultural inheritance, Wuhu Iron Paintings are a symbol of local culture, urban development, and people’s spirit, and the production of Wuhu Iron Paintings requires constant forging and shaping of iron and steel in order to build a complete picture.

#### 2.1.2. Extraction of Artistic Characteristics of Wuhu Iron Paintings in China

In view of the limitations of style migration, quantifiable attributes should be chosen for the extraction of stylistic features for Chinese Wuhu Iron Paintings. In addition, for Wuhu Iron Paintings, the style migration should not only ‘take shape’, but, more importantly, ‘convey the spirit’, so that users can directly distinguish the specific source of style features. The specific style features are extracted as shown in the Table 1.

### 2.2. Image Style Transfer

Image Style Transfer serves as an important research direction in the field of computer vision [25,26,27,28,29]. Its goal is to preserve the structure of the content image, while giving it the artistic style of another image. With the advent of the deep learning era, Image Style Transfer methods have evolved; for example, the traditional methods of [10] have transitioned to the iterative paradigm-based [11] and feed-forward network-based [12,13] in development. Gatys et al. [9] first applied convolutional neural networks (CNNs) to style transfer by iteratively optimizing noisy images to achieve stylization. Flow-based ArtFlow [17] with Projected Flow Network (PFN) achieves unbiased content results, while IEST [18] and CAST [19] achieve attractive results by using contrast learning.

Although the current progress has made great advances for generic style and Western oil painting style transfers, based on the inherent technical differences [30,31,32,33,34,35,36] between Chinese painting and Western oil painting, it is difficult to achieve a better balance of generic style for better artistic creation. Therefore, Ref. [37] proposed a style shift towards Chinese painting with unique ink characteristics. However, the existing methods still face great challenges due to the more three-dimensional layering and more complex techniques of Chinese Wuhu Iron Paintings.

### 2.3. Vision Transformer for Image

Transformer was originally designed for natural language processing tasks [38], but due to its unique architecture and representation capabilities, it was introduced to the field of computer vision [39,40,41,42,43,44,45,46]. Meanwhile, in order to alleviate the high computational cost problem faced by the Vision Transformer, DeiT [47] improves the original ViT by using methods such as strong data augmentation to eliminate the reliance on large amounts of data. Vision Transformer has also shown a strong impact in the field of style transfer [48,49,50,51,52]. In [53], a novel axial attention transform encoder was developed, which significantly improves the operational efficiency in style transfer tasks.

Recently, there has been a surge of interest in transformers for hierarchical architectures (LeViT [49] and CvT [54]). StyA2K, proposed by Zhu et al. [55], shows exceptional performance in preserving semantic structure and presenting consistent style patterns. Ref. [16] achieves a powerful generic style transfer by utilizing output merging of different windows’ attention. However, for Wuhu Iron Paintings, a traditional Chinese art, these methods, although they can encode feature information well, are unable to distinguish important and redundant features well for separation and decoding in the transfer process, resulting in unsatisfactory converted images.

## 3. Methodology

**Figure 2:**
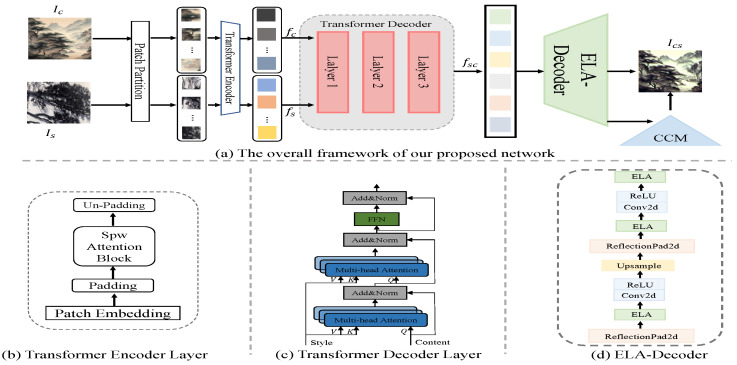
(**a**) demonstrates the structure of our framework. $I_{c},I_{s}$ denote content image and style image, respectively. $f_{c},f_{s}$ are the features extracted for content and style images. $f_{sc}$ denotes the transformed features obtained through the decode. (**b**) demonstrates the Transformer Encoder Layer. CCM denotes the Content Correction Module. (**c**) demonstrates the Transformer Decoder Layer. (**d**) demonstrates the ELA-Decoder.

Inspired by [16], We introduced the Hierarchical Vision Transformer, and effectively improved its decoder and detail processing. In this paper, our goal is to explore a style transfer network for Chinese Wuhu Iron Paintings that leads to perception-oriented transfer. As shown in Figure 2, we first decompose the style image and the content image based on multi-level Window Attention, and then encode the feature using a transformer encoder. Subsequently, a transformer decoder is utilized to decode the features initially, and the Efficient Local Attention Decoder is designed to focus on the style features and important content features to achieve effective decoding of the abstract style. In addition, the decoded features are fed in parallel to the content correction module to reject redundant features further and ultimately achieve fidelity transfer results. In the following sections, we will gradually elaborate on the proposed method in detail.

### 3.1. Hierarchical Visual Transformer

**Figure 3:**
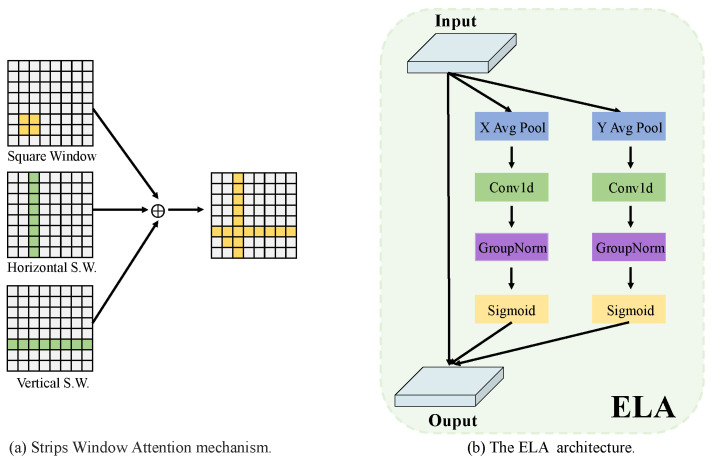
(**a**) shows the computation of Strips Window Attention. (**b**) shows the structure of Efficient Local Attention.

We first divide the content and style images into patch blocks of size 2 × 2 non-overlapping each other using the patch partitioning module. Subsequently, we deliver them to an encoder consisting of Strips Window Attention [16], and fill operations via linear embedding. Specifically, the fill operation is applied to achieve integer division filling for multiple windows. As shown in Figure 3a, Strips Window Attention consists of three different Window Attentions, where the horizontal and vertical Strip Windows pay attention to capture the longwall information and relevance of the feature, and the square window’s attention is focused on the surrounding information. An effective balance between short-range and long-range dependencies is achieved by integrating information from different windows [16] to expand the target’s sensory field. For Window Attention, we follow the previous work [16,48,56] for the calculation of the relative position deviation B, i.e.,

(1) $$W-MSA_{M\times M}\left(Q,K,V\right)=Softmax\left(\frac{QK^{T}}{\sqrt{d}}+B\right)V,$$

where *Q*, *K*, and *V* are the query, key, and value matrices, *d* is the matrix dimension, and $W-MSA_{M\times M}$ denotes the polytope self-attention using an M×M window.

Meanwhile, to achieve a multi-scale fusion of features during the encoding process, we extract elements at two-step intervals along the horizontal and vertical axes by merging the patches as a downsampling module. After feature encoding using the Strips Window Attention encoder, we perform the style transfer process through a multilayer Transformer decoder. Specifically, we perform inter-feature attention using MSA at each multi-head attention and use LayerNorm afterward. Finally, we output the migrated features through Feed-Forward Network (FFN) and LayerNorm.

### 3.2. ELA-Decoder Module

Previous studies have been able to capture features well and perform the initial transfer, but have not achieved good results when decoding migrated features. Particularly in the case of works of art with complex lines, such as Iron Paintings, previous approaches have scaled back style-specific concerns in order to accommodate generic styles. Thus, for the decoding process, effective attention to the complex features is lacking, thus prompting us to introduce effective localized attention to mitigate this challenge. As shown in Figure 2d, we utilize an effective combination of VGG and Efficient Local Attention to focus on key regions of decoded features and select abstract style and content features with adaptive relevance for more efficient style feature transformation.

We embed the Efficient Local Attention Block into the VGG network to extract the features of the key parts more efficiently, and to generate style images with more details. Specifically, we feed the output of the feature by the transformer decoder into ReflectionPad2d Block, and then we feed the output into the Efficient Local Attention (ELA) Module. The ELA module is shown in Figure 3b. First, the input features are pooled using strip-pooling to capture long-distance dependencies in the spatial dimension, followed by a convolution operation to process the sequence signal, which is more adept at processing sequence signals and lighter compared to 2D convolution. The operation is defined as follows:

(2) $$y^{h}=\varrho \left(G_{n}\left(F_{h}\left(Z_{h}\right)\right)\right),y^{w}=\varrho \left(G_{n}\left(F_{w}\left(Z_{w}\right)\right)\right),$$

where $F_{h}$ and $F_{w}$ denote one-dimensional convolutions and ϱ denotes a nonlinear activation function.

We multiply the obtained vectors in the horizontal and vertical directions with the input vectors to obtain the output, which is defined as follows:

(3) $$Y=x\times y^{h}\times y^{w},$$

After obtaining the output, we perform the Conv and ReLU operations, and then upsample the features with the same resolution size as the input image. To make the details of the image fit the style image better, we loop into that network structure again, but without the upsampling operation.

### 3.3. Content Correction Module

**Figure 4:**
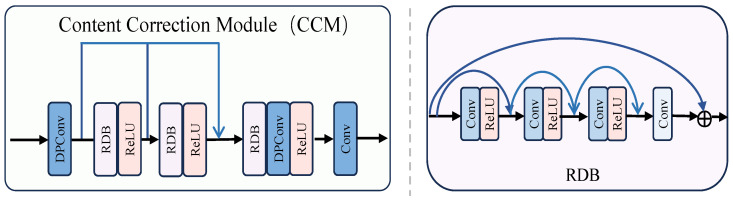
The architecture of the Content Correction Module.

We implement a Content Correction Module, as shown in Figure 4, to further process redundant features using a residual dense architecture combined with depth separable convolution. Specifically, we first extract shallow information through a layer of depth-separable convolution [57,58]. Subsequently, the combination of Residual Dense Block (RDB) [59] with four layers of residual dense architecture and ReLU is used to refine the features further and fuse all the information to enhance the memory of shallow features to deeper ones. Finally, we reconstruct noisy mappings and redundant features by combining deep separable convolution and ReLU. By removing the learned noise mappings, we further achieve effective style transfer. The operation is defined as follows:

(4) $$I_{cs}=A_{D}\left(f_{cs}\right)-A_{D}\left(CCM\left(f_{cs}\right)\right),$$

where $A_{D}$ is the attention decoder, $f_{cs}$ is the output feature, and CCM denotes the content correction operation.

### 3.4. Network Training

**Figure 5:**
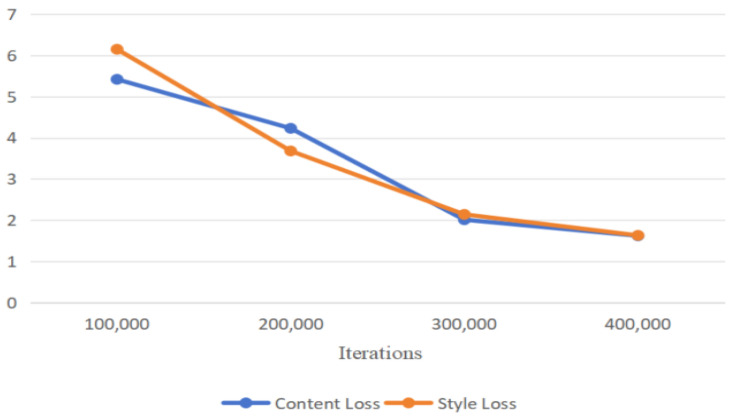
The content loss and style loss during the training process vary with the number of iterations.

We measure the content difference between the stylized image $I_{cs}$ and the content image $I_{c}$ by content loss, the content loss is the Euclidean distance between the mean-variance channel-wise normalized target features, and the mean-variance channel-wise normalized features of the output image VGG19 features, defined as

(5) $$L_{content}=\sum_{5}^{l=4}{∥\Phi_{image}^{l}\left(I_{cs}\right)-\Phi_{image}^{l}\left(I_{c}\right)∥}_{2},$$

where $\Phi_{image}$ denotes that the image encoder extracts features from the pre-trained VGG19 model, and *l* is the set of features consisting of the fourth and fifth layers in VGG19.

(6) $$L_{content}=\sum_{5}^{l=4}\beta_{1}{∥\Phi_{image}^{l}\left(I_{cs}\right)-\Phi_{image}^{l}\left(I_{s}\right)∥}_{2},$$

where $\beta_{l}$ is the weight of the feature loss in layer *l* of the VGG19 model. To consider both the global statistics and the semantically local mapping between the content features and the style features, we also use identity loss [60] to further preserve the structure of the content image and the stylistic features of the style image. The two different identity losses are defined as

(7) $$L_{id1}={∥I_{cc}-I_{c}∥}_{2}+{∥I_{ss}-I_{s}∥}_{2},$$

(8) $$L_{id2}=\sum_{l\in N}{∥\Phi_{l}\left(I_{cc}\right)-\Phi_{l}\left(I_{c}\right)∥}_{2}+{∥\Phi_{l}\left(I_{ss}\right)-\Phi_{l}\left(I_{s}\right)∥}_{2},$$

where $I_{cc}$ (or $I_{ss}$) denotes the output image stylized from two images of the same content (or style). Therefore, the total loss function is defined as

(9) $$L_{total}=\lambda_{c}L_{content}+\lambda_{s}L_{style}+\lambda_{id1}L_{id1}+\lambda_{id2}L_{id2},$$

where $\lambda_{c}$, $\lambda_{s}$, $\lambda_{id1}$, and $\lambda_{id2}$ are the weights for different losses. We set the weights to 2, 3, 50, and 1 to mitigate the effect of magnitude differences. The variation in content loss and style loss with the number of iterations during training is shown in Figure 5.

## 4. Experiment

### 4.1. Implementation Details

We used PyTorch (https://pytorch.org/) to implement our framework on two NVIDIA RTX 3090 GPUs. Under limited experimental conditions, the network uses the Adam optimizer, and through experimental comparison and evaluation, it is found that the initial learning rate is set to 1$\;\times \;10^{-4}$ and the batch size is set to 8. In addition, we first use MS-COCO [61] and WikiArt [62] as the content dataset and the style dataset, respectively, for initial training. Then, we used the Wuhu Iron Paintings dataset for fine-tuning and validation.

### 4.2. Comparison Experiment

#### 4.2.1. Qualitative Comparison

**Figure 6:**
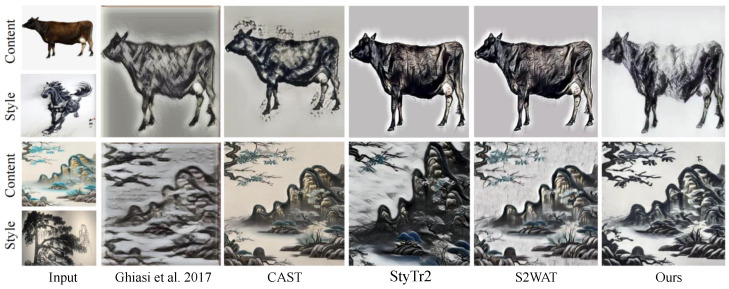
Visualization results of landscape paintings and flora and fauna on the Chinese Wuhu Iron Painting dataset. We compare the effects between our method and previous SOTA methods Ghiasi et al. [63], CAST [19], StyTr2 [48], and S2WAT [16].

**Figure 7:**
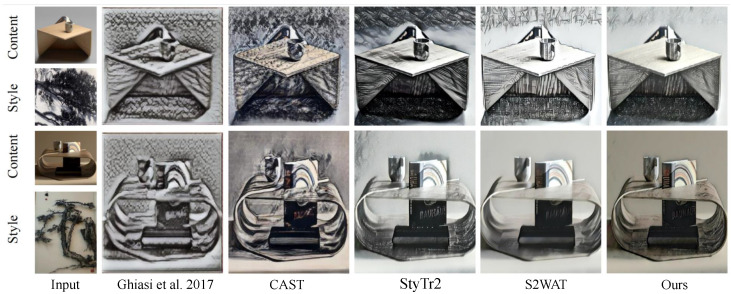
Visualization results of Furniture Types on the Chinese Wuhu Iron Painting dataset. We compare the effects between our method and previous SOTA methods Ghiasi et al. [63], CAST [19], StyTr2 [48], and S2WAT [16].

**Figure 8:**
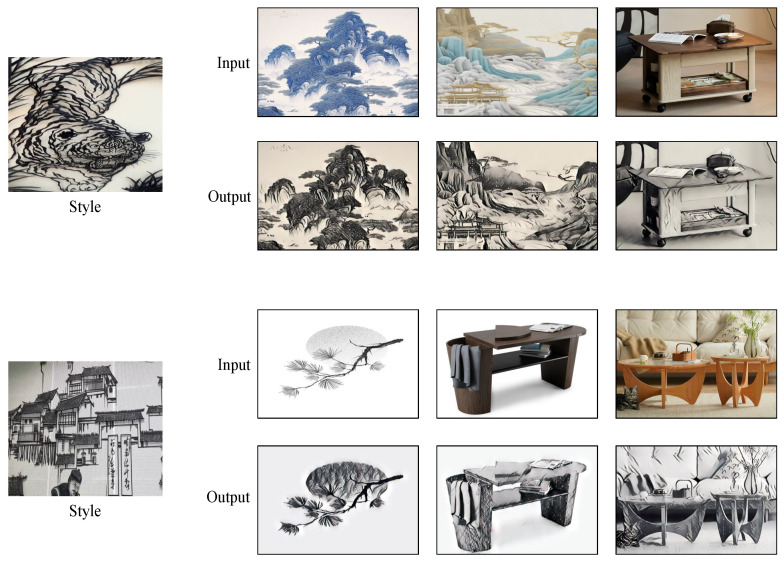
Partial visualization results of Wuhu Iron Paintings style transfer.

In this section, we compare the effects between the StWip and previous SOTA methods Ghiasi et al. [63], CAST [19], StyTr2 [48], and S2WAT [16]. Figure 6 presents the visualization outcomes of landscape and flora and fauna on the Chinese Wuhu Iron Paintings dataset, showing improved results due to similarities with the dataset’s characteristics. These improvements are particularly evident in line smoothness, clarity, metallic texture, outline definition, black-and-white contrast, and three-dimensional artistic expression. However, the current method fails to capture the stylistic nuances of Wuhu Iron Paintings, leading to significant style deviations. CAST maintains content structure, but misses complex style patterns, causing style corruption. StyTr2 and S2WAT, while achieving good color results through hierarchical visual transformers, lack local and global detail, failing to capture Iron Painting line details. In contrast, our method excels in preserving the stylized details of Wuhu Iron Paintings. Figure 7 demonstrates the challenges of applying this style to furniture, especially with complex layered paintings. The SOTA approach results in excessive features and shadows, whereas our content correction module, tailored for Wuhu Iron Paintings’ complexity, significantly reduces redundant features and shadows, showing effective results. As shown in Figure 8, we show the partial visualization results.

#### 4.2.2. Quantitative Comparison

In this section, we utilize losses as an indirect indicator. As shown in Table 2, compared to other methods, the content and style loss of our proposed method obtains the lowest value, which verifies the effective migration performance to the Wuhu Iron Painting dataset. Meanwhile, to ensure the validity of the comparative experiment, a quantitative analysis was conducted in the form of questionnaire voting. A total of 200 subjects were invited to this experiment, of which a total of 178 valid votes and 22 invalid votes were cast, with an effective voting rate of 89%. The specific voting results are shown in Table 3.

Combined with the results in the above table, it can be seen that the style conversion method designed in the article obtains the highest number of votes in the voting for different types of effect images, which shows that it is the most effective and most recognized by the users in converting the style of Wuhu Iron Paintings. In the voting of animal, landscape, and product categories, the method designed in the article obtained high votes, especially in the voting of sample image 1 of the houseware category; the user votes reached 79, which shows its wide applicability and high efficiency in dealing with various types of images. This result also reflects the technical superiority of the designed method, which is able to retain the original features of the image better and successfully convert it to the Wuhu Iron Paintings style.

### 4.3. Ablation Study

#### 4.3.1. ELA-Decoder Module

**Figure 9:**
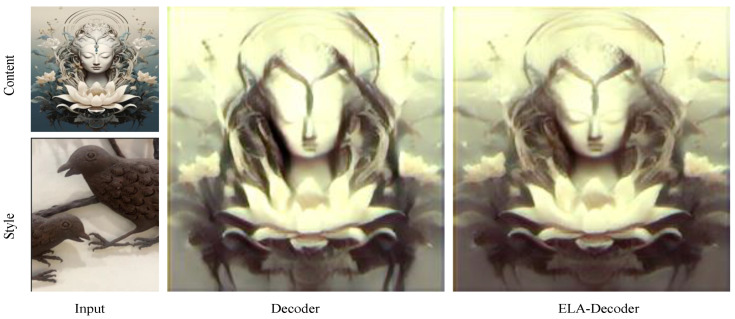
ELA-Decoder Module for ablation experiments. Decoder indicates the use of a basic VGG setup.

In order to demonstrate the effectiveness and superiority of the ELA-Decoder Module, we conducted experiments to test it using the decoder designed with [16]. The results are shown in Figure 9. Due to the lack of attention to the local features and the dependence on the overall features, the conversion result fails to effectively capture the hierarchical and line features of the Chinese Wuhu Iron Paintings. This effectively validates the superiority of the ELA decoding module, which provides excellent decoding of both local and overall features.

#### 4.3.2. Content Correction Module

**Figure 10:**
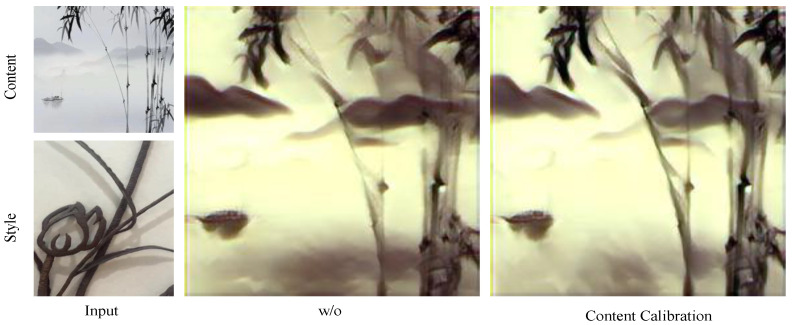
Ablation experiments with content correction module; w/o indicates that no content correction module was used.

The effectiveness of the content correction module designed using the dense residual architecture. As shown in Figure 7, the effectiveness has been initially verified in furniture type presentation. In addition, we further experimentally verify its superiority. As shown in Figure 10, we demonstrate the visual effect without the content correction module, and it can be seen that redundant features are obviously increased in the conversion results without the content correction module compared to the full setup. This further confirms our conjecture.

### 4.4. Expert Scoring Experiment

In order to verify the effectiveness of the methodology proposed in the article, this study empirically evaluated the method using the expert scoring method, inviting 60 teachers and students from the fields of non-heritage culture, fine arts, and design, as well as non-heritage inheritors, as participants. The core of the experiment is to comprehensively evaluate the effectiveness of the method in style transformation through the subjective experience of the participants, from the presentation of smooth lines, the clever use of black and white contrast, the realistic simulation of metal textures, and other key dimensions. We performed this by making the original image and the converted style image available together for the experts’ evaluation. This not only tests the artistic integration ability of the technical means, but also explores its potential value in the inheritance and development of intangible cultural heritage, providing a strong empirical basis for the modernization and transformation of traditional crafts.

A total of 60 questionnaires were collected in this study; 4 invalid questionnaires were screened and eliminated, and the final valid questionnaires amounted to 56, with a validity rate of 93.33%. In the experimental session, we randomly selected 4 groups of image samples processed by the style conversion technology as test subjects. Each participant, based on their personal subjective experience, rated the style migration effect and aesthetics of each group of samples on six dimensions: smoothness of lines, metal texture performance, simplicity of outline, black and white contrast effect, three-dimensional depth of the image, and the art of leaving white space. The scoring was based on a five-point scale, with 1 being the lowest and 5 being the highest, and the subjects gave the corresponding scores for the style migration effect and aesthetics of each image sample according to their subjective feelings. In this paper, a reliability analysis was conducted, and the results showed that Cronbach’s α coefficient reached 0.931, which fully proved the high reliability of the scoring items and provided solid data support for the subsequent research. The specific experimental results are shown in Table 4.

## 5. Analysis of Design Application Based on the Conversion of Wuhu Iron Paintings Style

Path 1: Product design applications. The application of Wuhu Iron Paintings style conversion method in product design is not only a cross-border cooperation of art forms, but also a model of deep integration of traditional and modern design. Our methodology refines and analyzes the unique artistic features of Chinese Wuhu Iron Painting, such as its iconic smooth lines, metallic expression, contrasting black and white effects, etc., and through the combination of modern design thinking and high-tech means, the converted effects are cleverly and quickly integrated into modern product design, bringing more possibilities to the original product design. This process involves the cross-fertilization of multiple disciplines such as computer science, design, craft aesthetics, etc. Designers can make use of modern technologies such as 3D modeling, laser cutting, precision casting, etc., to quickly and accurately reproduce the artistic charm of Iron Paintings, and at the same time, give the products a brand-new form and function. The use of this design method not only greatly broadens the creative space of product design, but also provides a strong technical support for the transformation of traditional art product design.

**Figure 11:**
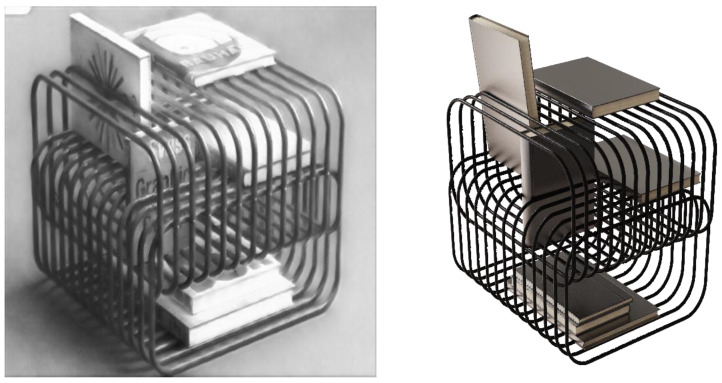
Application effect of Wuhu Iron Paintings style conversion method in product design and 3D modeling effect.

As shown in Figure 11, a modern minimalist furniture product was chosen as the base for applying our style conversion method, successfully integrating Wuhu Iron Painting elements. Rhino software7.0 facilitated 3D modeling based on the converted style, while KeyShot12 was utilized for rendering, accurately simulating the metallic texture of Wuhu Iron Painting by fine-tuning material properties. The visual effect was enhanced with strategic lighting and background settings, particularly emphasizing the black-and-white contrast to add depth and realism to the model. The KeyShot rendering outcome not only highlights the distinctive appeal of the Wuhu Iron Painting style, but also confirms the efficacy of our style conversion approach and the 3D modeling and rendering techniques employed.

Path 2: Environmental design applications. Wuhu Iron Painting has long occupied an important place in the field of decorative arts with its unique forging techniques and aesthetic interests, and the application of its style conversion method provides a rich source of material and inspiration for environmental design. In the current space construction, it can be analyzed from the following four dimensions through the style conversion method.

**Figure 12:**
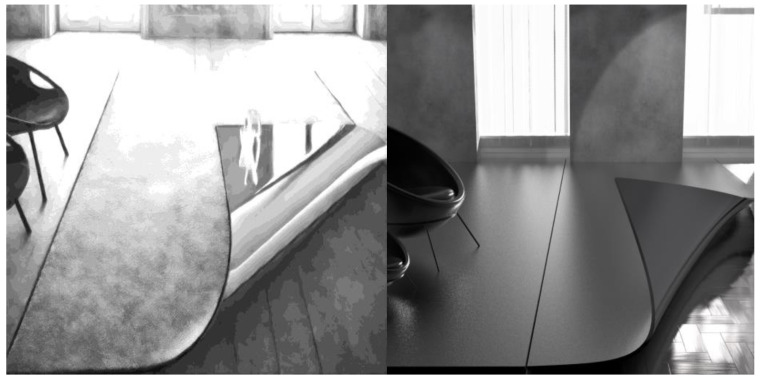
The effect of Wuhu Iron Painting style conversion method in environmental design and 3D modeling effect.

The Wuhu Iron Painting style’s application in modern design achieves several objectives. It rapidly adapts to interior spaces, transforming elements like partitions and walls, and enhancing the artistic appeal of modern interiors. In landscape and public art, this style offers innovative design solutions, integrating Wuhu Iron Painting’s artistic language into sculptures and water features, thereby enriching the landscape’s visual hierarchy and cultural significance. Additionally, it facilitates spatial division and creates visual focal points, diversifying the artistic expression of space. The transformation method, demonstrated through 3D modeling with Rhino and rendering with Keyshot, maintains functionality while significantly improving the space’s artistic and cultural ambiance, validating its effectiveness in contemporary design. As shown in Figure 12, the original space design sample was transformed into Wuhu Iron Painting style to generate a new style case rich in traditional cultural elements. Subsequently, Rhino software was used for 3D modeling, and Keyshot for rendering, to present the transformed visual effect. By comparing the original and transformed design effects, it is found that the transformation method not only preserves the functionality of the space, but also significantly enhances its artistic expression and cultural atmosphere, which strongly verifies the value and effectiveness of the transformation method of Wuhu Iron Painting style in modern space design.

**Figure 13:**
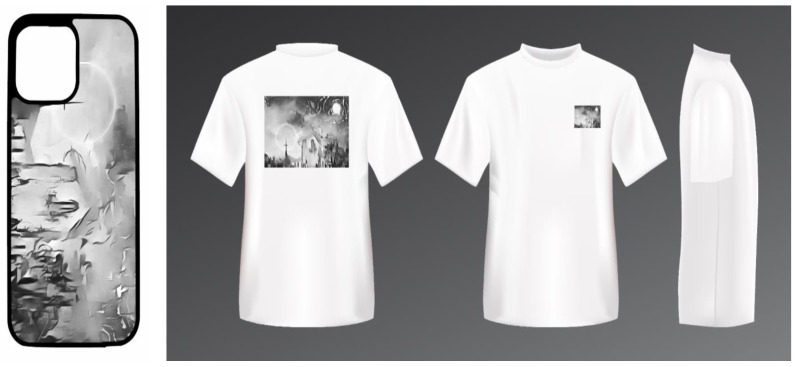
The effect of Wuhu Iron Paintings style conversion method in the application of digital application.

**Figure 14:**
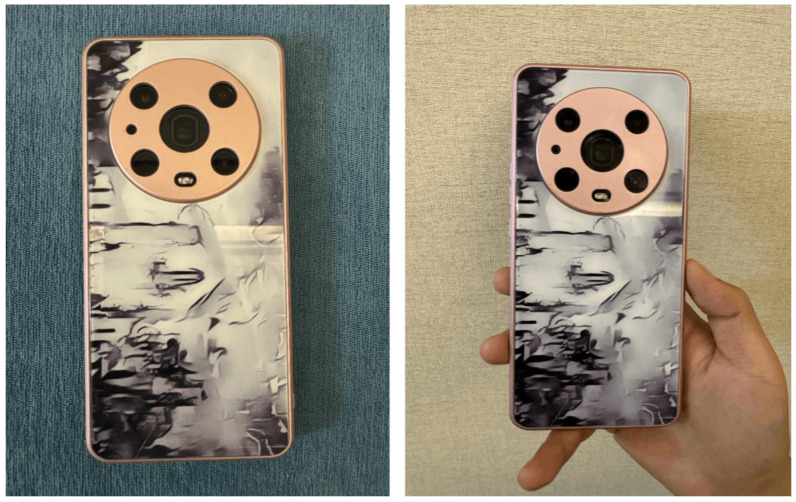
Practical product results for digital applications.

Path 3: Digital application. The digital application for Wuhu Iron Painting involves creating a virtual exhibition space using VR and style conversion methods. This space lets users instantly change their environment to one that displays the Wuhu Iron Painting style through simple actions like a button press or gesture interaction, providing a global audience with an immersive art experience. Additionally, a digital interactive platform is built that integrates style conversion with digital printing. Users can select a style sample, convert it with one click, and then print the design onto various items like clothing and home decor. This approach enhances the art’s reach, encourages public engagement in its preservation and innovation, and involves more people in supporting this traditional art form. As shown in Figure 13 and Figure 14, in practice, by integrating the style conversion algorithm and digital printing technology, users can quickly generate the design effect of the Wuhu Iron Painting style and print out the physical object for use instantly. This process not only accelerates the transformation from design to object, but also effectively enhances the users’ intuitive perception of the charm of Wuhu Iron Paintings.

In summary, through the method of converting the style of Wuhu Iron Painting, the style of Iron Painting can be migrated to various objects and environmental spaces, injecting new vitality and cultural connotation into modern design. This kind of inheritance and innovation not only helps to protect and promote the art of Iron Painting, but also provides new ideas for the inheritance promotion, and application of other intangible cultural heritage.

## 6. Discussion

In this section, we will provide a detailed introduction to the Wuhu Iron Paintings dataset, and discuss the limitations of our method.

### 6.1. Wuhu Iron Paintings Dataset

**Figure 15:**
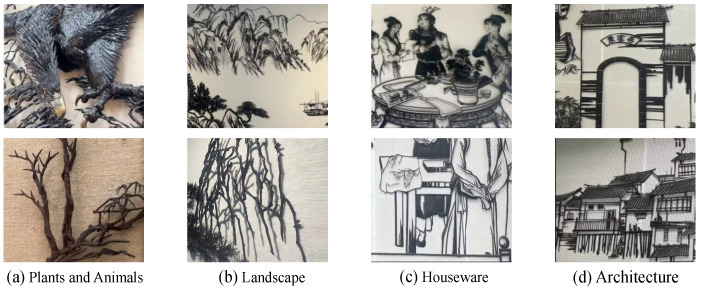
A visualization of the various categories of the Wuhu Iron Paintings dataset.

We have collected 500 Wuhu Iron Paintings from the real world and categorized them into five major classes: plants and animals, landscapes, houseware, and architecture. An example of the classification of the Wuhu Iron Paintings dataset is shown in Figure 15. The plants and animals category features vivid depictions of animals and plants with intricate textures created through techniques like hammering, bending, and welding. The landscape category portrays natural scenery such as mountains, trees, and rivers, using iron texture and lines to create depth. The houseware category includes furniture and decorations, blending practicality with artistry. The architecture category showcases traditional buildings, such as pavilions and bridges, with precise structural depictions and fine details.

### 6.2. Limitation

**Figure 16:**
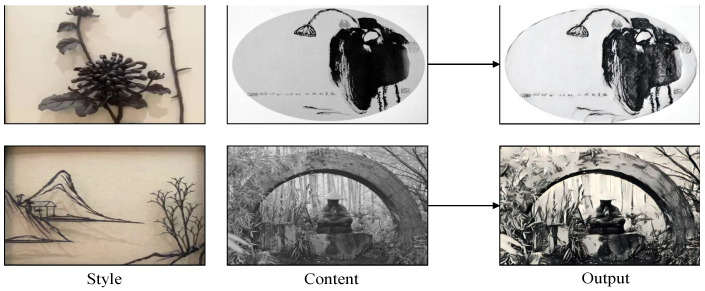
The limitations of our method. When the style image and content image are too similar or when the content image contains overly complex details, our method still encounters certain challenges.

Although our method has made significant progress in transferring the style of Wuhu Iron Paintings, there are still some limitations that need to be addressed. As shown in the first row of Figure 16, when the style image and content image are overly similar, the network struggles to effectively decouple content and style features during the transformation process, resulting in suboptimal visual outcomes. Additionally, as depicted in the second row of Figure 16, when using a relatively simple style image for a content image with a complex background, feature coupling may introduce deviations, leading to unnecessary artifacts.

## 7. Conclusions

This paper presents an efficient style transfer network for Chinese Wuhu Iron Paintings, built on a Hierarchical Vision Transformer framework to achieve image style transformation. The network employs an encoder with a Strips Window Attention mechanism for efficient feature encoding, combined with a specially designed attention decoder and content correction module to harmonize content and style features. This ensures vivid stylistic expression and rich content preservation during the style transfer process. Extensive experimental results demonstrate that our method achieves state-of-the-art performance on the Wuhu Iron Paintings dataset. Compared to existing advanced methods, our approach achieves the lowest content loss and style loss, at 1.62 and 1.63, respectively. In qualitative analyses, expert evaluations further validated our method, with our approach receiving the highest number of votes, underscoring its outstanding performance. Additionally, the paper explores applications of Wuhu Iron Paintings in product, environmental, and digital design, providing a novel approach to the preservation and innovation of intangible cultural heritage while offering new directions for future development in this field.

## Figures and Tables

**Table 1:** Wuhu Iron Painting style characteristics extraction table.

Type	Specific Features	Feature Description
Modeling	Flowing line	Unlike other images, Wuhu Iron Paintings are made of steel title, focusing on the expression of modeling lines.
	Brilliance	The material of Wuhu Iron Paintings is metal, which has a strong metallic luster, which is different from the data used in the current field.
	Simple outline	In the production of Wuhu Iron Paintings, the shape of the object will be highly generalized, forming a brief and condensed stylistic imagery.
Color	Black and white	In terms of color, Wuhu Iron Paintings inherit the characteristics of Chinese painting, often with white as the background, and black lines for the composition of the painting, to create a strong contrast between black and white, and with the Chinese painting of the color of the beloved ink it coincides with.
Hairstyle	3D artistry	Wuhu Iron Paintings is a three-dimensional painting with a unique three-dimensional texture, and its three-dimensionality is mainly divided into two kinds: one is the height of the object itself, and the other is the level of interspersed between the objects. The data used in the current field are mostly flat, which also shows that it is challenging for us to migrate the style of Wuhu Iron Paintings.
	Leave a blank page	Wuhu Iron Paintings, in terms of the white pictures from the influence of Chinese painting, have specific whites, divided into the following four kinds: one is the composition of white, two is the mood of white, three is the reality of white, and four is the scene of white. This situation is also one of the difficulties we face, because Western art images do not have this type of art.

**Table 2:** Qualitative results of furniture types on the Chinese Wuhu Iron Paintings dataset.

Type	CAST	StyTr2	S2Wat	WCT	Ours
Content Loss ↓	2.17	1.91	1.67	2.56	1.62
Style Loss ↓	4.43	1.67	1.75	2.23	1.63
Time(seconds) ↓	0.042	0.237	0.558	0.590	0.573

**Table 3:** Comparison of experimental output effect map voting result statistics. The red font indicates that it received the most votes.

Methods	Ghiasi et al. [63]	CAST	StyTr2	S2WAT	Ours
Animal species sample	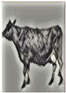	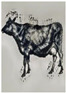	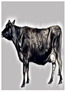	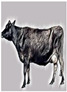	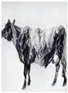
	19	47	25	18	69
Landscape sample	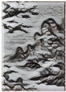	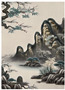	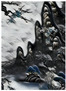	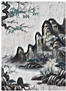	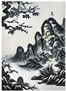
	11	28	35	33	71
Houseware -1	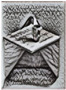	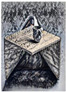	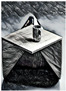	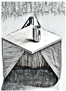	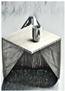
	16	26	41	16	79
Houseware -2	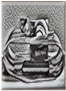	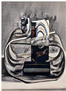	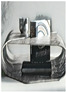	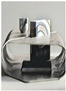	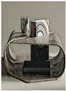
	14	34	46	29	54

**Table 4:** Table of expert scoring and assessment results.

Type	Samples			
	**Animal Samples**	**Landscape Sample**	**Houseware Sample**	**Houseware Samples**
	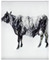	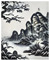	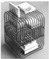	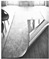
Line fluidity	3.98	4.27	4.02	4.18
Metallic expression	4.16	4.07	4.29	4.30
Contour simplicity	3.91	4.11	4.23	4.25
Black and white contrast effect	4.25	4.40	3.98	4.14
Stereoscopic depth of picture	4.35	4.14	4.25	4.30
Leave a blank page	4.09	4.21	3.96	4.17
Aggregate score	4.12	4.20	4.12	4.22

## Data Availability

The data presented in this study are available on request from the corresponding author. The data are not publicly available due to copyright issues.
